# Methylprednisolone for COVID-19 Patients Admitted to a Tertiary Care Hospital: A Single-Centre Study

**DOI:** 10.7759/cureus.17693

**Published:** 2021-09-03

**Authors:** Bilal Ahmad, Adnan Manzar, Saba Khrshid, Naeem Ul Hassan, Anjum Muhammad

**Affiliations:** 1 Endocrinology and Diabetes, Pak-Emirates Military Hospital Rawalpindi, Rawalpindi, PAK; 2 Internal Medicine, Pak-Emirates Military Hospital, Rawalpindi, PAK; 3 Internal Medicine, Combined Military Hospital, Rawalpindi, PAK; 4 Dermatology, Pak-Emirates Military Hospital, Rawalpindi, PAK

**Keywords:** methylprednisolone, corticosteroids in covid-19, covid-19 treatment, survival outcome covid-19, cytokine release syndrome (crs)

## Abstract

Background: The role of various corticosteroids in the management of COVID-19 is evolving. Following an initial lack of evidence, the relatively novel data, supporting the survival benefit to severe and critical COVID-19 patients, is of limited scale.

Materials and methods: This retrospective study observed medical records and disease outcomes of 200 patients with moderate, severe and critical COVID-19 receiving methylprednisolone (MP). The dose of methylprednisolone was 0.5 to 2 mg per kg in these patients.

Results: Median age of presentation was 59 years. The median duration of symptoms at presentation was five days. The most common presenting symptoms were cough (77.5%), fever (67.5%) and shortness of breath (63.5%). Majority of patients (85%) presented in the first week of illness. One or more comorbidities were present in 75% of patients. Complications seen in the study cohort were cytokine release syndrome (CRS) 92 (46%), acute respiratory distress syndrome (ARDS) 44 (22%) and multi-organ dysfunction 17 (8.5%). The median time for initiation of corticosteroid therapy was four hours. Overall survival (OS) in patients receiving methylprednisolone was 83.5%. The OS for patients with moderate, severe and critical diseases was 97.8%, 86.2% and 62%, respectively (p<0.001).

Conclusion: Steroids like methylprednisolone are useful in COVID-19 admitted patients and provide excellent survival outcomes.

## Introduction

Till July 2021, severe acute respiratory syndrome coronavirus 2 (SARS-CoV2) has affected more than 18 million people globally and 0.9 million people in Pakistan [1]. Despite continuous research and clinical trials, there is minimal success in establishing an effective treatment regimen against coronavirus disease 2019 (COVID-19). Treatment options beyond supportive care are limited, especially in developing countries. Trials of Remdesivir and Interleukin-6 inhibitors are in progress with limited outcomes [2]. After the initial viremic phase, the majority of complications associated with COVID-19 are attributable to immune dysregulation and cytokine release syndrome (CRS). CRS is an acute inflammatory syndrome characterized by fever and multi-organ dysfunction associated with raised inflammatory markers including C-reactive proteins (CRP) and Interleukin-6 (IL-6) which can result in acute lung injury and acute respiratory distress syndrome (ARDS) [3]. CRS is present in 30% of patients with mild and 76% of patients with severe COVID-19 infection [4]. Steroids halt the production of cytokines and thereby have a potent anti-inflammatory effect [5]. Their use is not confined to the intensive care unit (ICU) patients; the use of steroids in non-ICU COVID-19 patients resulted in a reduced transfer to ICU, lower intubation rates and a decrease in mortality [6]. Methylprednisolone (MP) and dexamethasone have been in use for the treatment of COVID-19 patients, for the past one and half years with proven benefits. MP has shown better results in hospitalized patients [7]. This retrospective study aims to report the outcome of hospitalized COVID-19 patients treated with MP in a tertiary care hospital in Pakistan.

## Materials and methods

This retrospective study was carried out at the Department of Medicine, Pakistan Emirates Military Hospital (PEMH), Rawalpindi, Pakistan from April 1 to May 30, 2020. The study was approved by the hospital’s ethical review committee (A/28/103). Medical records of all the patients admitted with moderate, severe and critical COVID-19 disease, during the study period, receiving methylprednisolone were reviewed retrospectively. Additional inclusion criteria of the study included SARS-CoV2 positivity documented by polymerase chain reaction (PCR), use of methylprednisolone, patients of 20-80 years and both genders, hospital admission and at least 48 hours of steroids use. Exclusion criteria of the study were defined as the use of steroids other than methylprednisolone, death within 48 hours of admission, patients of haematological or solid organ malignancies, patients receiving other investigational drugs including Remdesvir, Tocilizumab, convalescent plasma, mesenchymal stem cells, plasma exchange and patients with mild COVID-19 disease. A total of 200 patients were enrolled in the study as per the above criteria. A confirmed case was defined as patients with a positive reverse transcriptase (RT) PCR for SARS-CoV2 in a nasopharyngeal specimen. Severity classification was done as per WHO criteria. The moderate disease was defined as COVID-19 positivity with <50% lung infiltrates and peripheral ground-glass opacities (GGOs) on the chest computed tomography (CT) scan but no evidence of hypoxemia on arterial blood gases (ABGs). Severe disease was defined as COVID-19 pneumonia with hypoxemia (respiratory rate >30/minute or partial pressure of O_2_ (PaO_2_) on ABGs <80 mmHg or lung infiltrates >50% of the lung field). The critical disease was defined as evidence of either respiratory failure (PaO_2_ < 60 mmHg) or multi-organ dysfunction syndrome (MODS) with Sequential Organ Failure Assessment (SOFA) score >10 or septic shock (systolic blood pressure (BP) less than 90 or less than 40 mmHg of baseline in hypertensive or urine output <0.5 ml/kg/hour). Treatment was given according to disease severity as per institutional guidelines for COVID-19 management. Patients with moderate disease received protocol-B (a combination of tablet aspirin 75 mg daily, injection enoxaparin 0.5 mg/kg once daily, methylprednisolone (MP) 0.5 mg/kg). Severe disease was treated with 1 mg/kg methylprednisolone, in addition to aspirin 150 mg and enoxaparin 0.5-1 mg/kg (protocol C). For patients with pulmonary infiltrates and hypoxia, awake prone posturing for 12-14 hours (when GGOs > 50%) and those unable to maintain oxygen saturation above 92% on room air, protocol D was used which was similar to protocol C except for the use of continuous positive airway pressure (CPAP) 8-10 cmH_2_O. Methylprednisolone 0.5 to 2 mg/kg was given to patients with CRS. Recovery was defined as an improvement of the patient’s condition from critical/severe to moderate or moderate to mild disease state, normalization of inflammatory markers such as CRP, lactate dehydrogenase (LDH), and serum ferritin levels, absolute lymphocyte count >1000 and prothrombin time (PT)/activated partial thromboplastin time (APPT) normalization. The primary endpointwas overall survival (OS). Outcome measures were death or discharge. Secondary outcome measures were median days of hospitalization and symptomatic improvement number of days to PCR negativity. Continuous variables were reported as median and range using student’s t-test or Mann-Whitney test as appropriate. Reporting of categorical data was done with the number (n) and percentage (%). The Chi-square test was used for the comparison of categorical variables. Kaplan-Meier survival analysis was done to analyze survival outcomes. Statistical analysis was done using SPSS version 23 (SPSS Inc., Chicago, IL).

## Results

The baseline demographic and clinical characteristics of the study cohort are shown in Table 1.

**Table 1:** Demographic and clinical characteristics of the study group

Characteristics	Total (n=200)
Median age (range)	59 (20–80)
Gender, n (%)	
Male	176 (88%)
Female	24 (12%)
Clinical features n (%)	
Fever	135 (67.5%)
Cough	155 (77.5%)
Shortness of breath	127 (63.5%)
Duration of symptoms at admission in days, median (range)	5 (1–15)
Laboratory values	
Absolute lymphocyte count × 10^9^/l, median (range)	1200(130–2400)
Platelet count × 10^9^/l, median (range)	181(60–450)
CRP (ug/ml), median (range)	61 (2–480)
D-dimers, median (range)	64 (200–1500)
Ferritin (ng/ml), median (range)	477 (36–8000)
ALT (IU/l), median (range)	41 (11–312)
LDH (U/l), median (range)	670 (160–2570)
Cardiac biomarkers, n (%) (troponins and Pro BNP)	
Normal	96 (48%)
Raised	104 (52%)
CT chest findings, n (%)	
Typical	137 (69%)
Indeterminate	19 (9.5%)
Atypical (consolidation, effusions and sub-pleural atelectasis)	22 (11%)
Normal	21 (10.5%)
Lung involvement, n (%)	
<50%	84 (42%)
>50%	94 (47%)
Normal	21 (11%)
Oxygen support type	
NIV	37 (18.5%)
IV	5 (2.5%)
Disease severity at presentation, n (%)	
Moderate	100 (50%)
Severe	58 (29%)
Critical	42 (21%)
Treatment protocol, n (%)	
Protocol B	72 (36%)
Protocol C	49 (24.5%)
Protocol D	79 (39.5%)
Day 7 PCR positivity, n (%) (n=170)	63 (37%)
Day 14 PCR positivity, n (%), n=(145)	20 (14%)
Discharge days, median (range)	11 (4–32)

**Figure 1:**
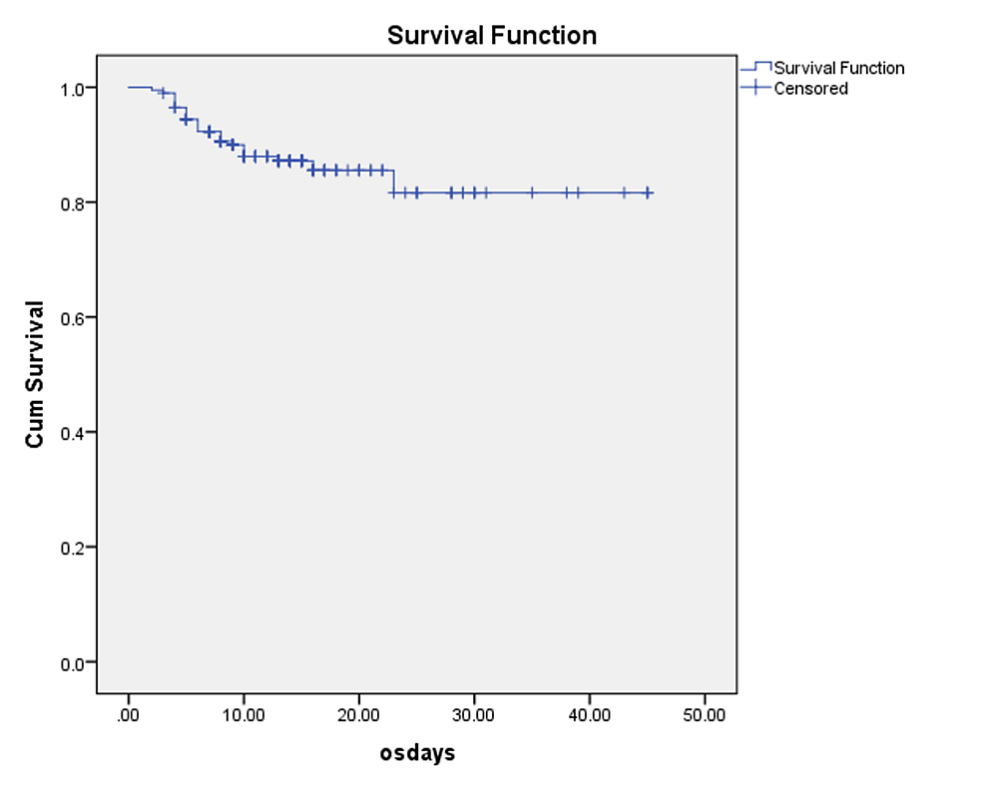
Overall survival was 87.4% in the study cohort

Median age at presentation was 59 years (range 20-80). Male female ratio was 8:1. Median duration of symptoms at presentation was five days (range 1-15). Cough 77.5% (n=155), fever 67.5% (n=135) and shortness of breath 63.5% (n=127) were the commonest presenting symptoms. Other symptoms included myalgia 23% (n=46), gastrointestinal symptoms 14.5% (n=29), vertigo 9% (n=18), headache 6.5% (n=13), anosmia 9.5% (n=19) and sore throat 6.5% (n=13). Majority of the patients 85% (n=170) presented within the first week of illness. One or more comorbidities were present in 75% (n=150) of the patients. Most common being hypertension in 35% (n=70), diabetes mellitus 31.5% (n=63), ischemic heart disease 22.5% (n=45) and chronic obstructive airway disease 9% (n=18). Laboratory abnormalities are summarized in Table 1. Cardiac biomarkers were found to be elevated in 52% (n=104) patients but did not correlate with the disease severity (p=0.08). Clinical symptoms and ECG changes of angina were seen in 5% (n=10), while myocardial infarction was seen in 3% (n=6) of the patients. CRS was the most common complication in 46% (n=92), followed by ARDS 22% (n=44) and multi-organ dysfunction in 8.5% (n=17) of our cohort. The median time for initiation of corticosteroid therapy was four hours (range 1-96 hours). Treatment protocol B (MP 0.5 mg/kg body weight) was used for moderate COVID-19 patients in 36% (n=72), protocol C (MP 1 mg/kg body weight) in 24.5% (n=49), while protocol D (MP 1 mg/kg body weight plus CPAP/invasive ventilation in 37% (n=74) of the cases. MP at a dose of 2 mg/kg body weight was administered in all 46% (n=92) of patients with CRS. Treatment escalation was needed in 12 patients where 2.5% (n=5) required invasive ventilation while 3.5% (n=7) required non-invasive ventilatory support. OS in study cohort was 83.5% (Figure 1).

**Figure 2:**
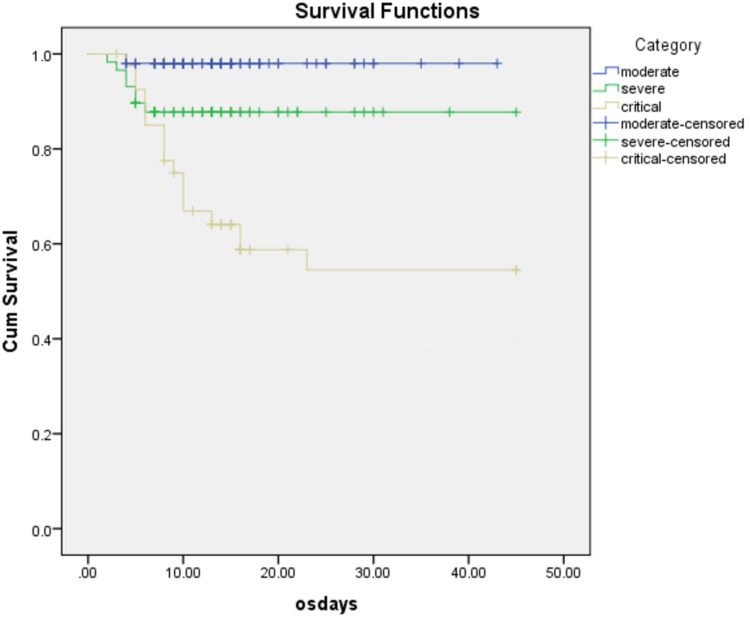
Overall survival for patients with moderate, severe and critical diseases was 97.8%, 86.2% and 62%, respectively, median 95% CI 11.07-34.92 (p<0.001)

OS for patients with moderate, severe and critical diseases was 97.8%, 86.2% and 62%, respectively (p<0.001; Figure 2).

## Discussion

Steroids have been in use since 1980 as potent anti-inflammatory agents for ARDS. Overproduction of pro-inflammatory cytokines and their dysregulation is the main cause of multi-organ dysfunction and ARDS in COVID-19. Methylprednisolone is a potent FDA-approved steroid since 1957, used in a variety of endocrine, rheumatological, dermatological, and vascular disorders. Its oral bioavailability is 89.9% [1]. Multiple studies have revealed its efficacy in reducing disease severity, hospital stays and the need for mechanical ventilation in the recent COVID-19 pandemic [8].

The mortality rate of hospitalized COVID-19 patients ranges from 20% to 37% [8,9]. The treatments options with proven efficacy for hospitalized COVID-19 patients are limited. Studies regarding the efficacy of Remdesivir are limited, moreover, availability and cost restrict its use in developing countries [2]. Conflicting data are available regarding the efficacy of tocilizumab [7], and convalescent plasma [8,9] while the role of therapeutic plasma exchange is being investigated [8]. Initial guidelines from the Infectious Disease Society of America advise against the routine use of corticosteroids for COVID pneumonia [10,11]. Moreover, the American Thoracic Society (ATS) does not recommend the use of corticosteroids in COVID-19. However, studies carried out by Ko et al. and Fadel et al. suggest the benefit of corticosteroids in COVID patients, particularly when used early in the disease course [5,12]. An interim report from the RECOVERY trial has documented survival benefits in patients receiving dexamethasone, thus paving way for further trials with corticosteroids in COVID-19 patients. This retrospective review reports the outcome of corticosteroids uses among COVID-19 patients in the South Asian population. With a median age of 59 years (range 20-80), patients in our cohort were younger as compared to the median age of 62 years in a study by Fadel et al. [12], while the median age in a study on the use of methylprednisolone by Wang et al. was 54 years (range 48-64 patients) [13]. The most common presenting symptoms in our study were cough, fever and SOB in 77.5%, 67.5% and 63.5% of the patients, respectively. Fadel et al. reported a similar presentation with cough (74.2%), fever (70.4%) and SOB (69.5%) in his study [12]. The median absolute lymphocyte count in our study was 1.2 × 109/l as compared to 0.86 × 109/l in a study by Wang et al. [13] and 0.88 × 109/l as reported in a study by Richardson et al. [14]. This difference can be due to different geographic locations and patient populations, warranting further studies. In our study, 50% (n=100) patients had severe or critical disease at the time of initiation of steroids as compared to 23% in a study by Fadel et al. [12]. Mortality in our cohort was 16.5% (n=33); 20% to 37% mortality rate has been documented in studies employing routine supportive care without corticosteroid use [14,15]. Fadel et al. reported a similar mortality rate (13.6%) in 132 patients treated with early steroids, while Wu et al. [16] reported 50% mortality among patients receiving methylprednisolone. Another study by Zhou et al. documented the benefits of low-dose steroids in COVID-19 [17]. Interim results of the RECOVERY trial documented dexamethasone as the first drug providing survival benefit in COVID-19 patients [18], although it was the only steroid used in this trial, and comparison with other steroids is not available. Although not directly comparable to our study, mortality in the RECOVERY trial was 21.6% compared to 16.5% in our research, further highlighting the effectiveness of methylprednisolone in COVID-19 patients. Our study has few limitations. Being a retrospective review, no control group is available. Methylprednisolone was used as per the institutional policy, hence the data for dexamethasone are not available. Different doses of MP were used as per the disease severity and no randomization was done to select the optimal dose.

## Conclusions

The world was exposed to unusual and unpredicted stress since the COVID 19 infection emerged. As the disease and its treatment modalities are still evolving we are in the continuous learning phase. Our study has provided evidence that Methylprednisolone and possibly other corticosteroids added to the standard care in hospitalized patients may result in better covid-19 disease outcomes. However, It is necessary to carry out randomized trials with different steroid formulations to document the efficacy and safety of corticosteroids in COVID-19 disease.
